# Integrating Cryo-Electron Microscopy and Molecular Dynamics Simulations to Investigate Membrane Binding of Influenza Virus Fusion Peptides

**DOI:** 10.1021/jacs.4c18441

**Published:** 2025-04-11

**Authors:** Piotr Setny, Paulina Borkowska, Remigiusz Worch

**Affiliations:** † Centre of New Technologies, University of Warsaw, 2C Banacha St., Warsaw, Poland 02-097, Poland; ‡ Nencki Institute of Experimental Biology, Polish Academy of Sciences, 3 Pasteur St., Warsaw 02-093, Poland

## Abstract

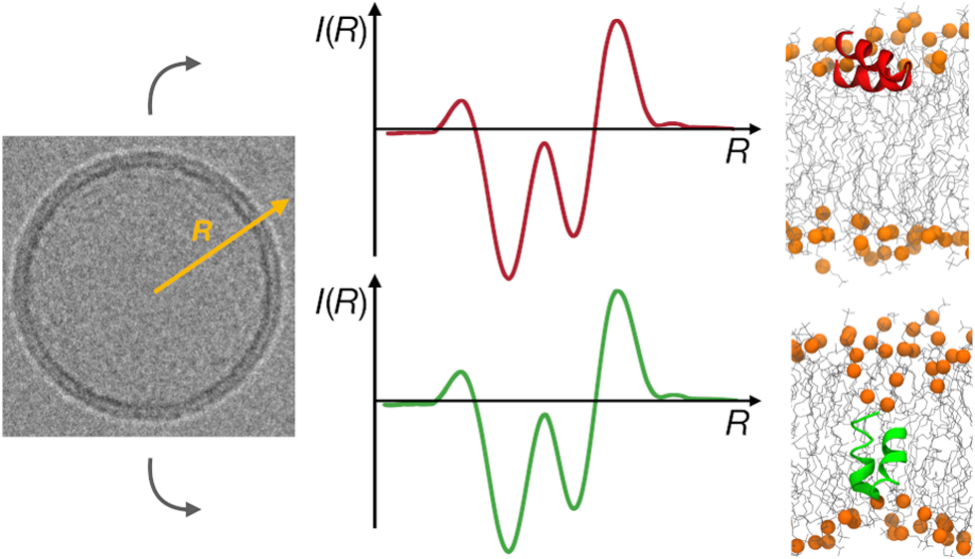

We propose an approach for determining the positioning of membrane-active peptides within a lipid bilayer. It is based on a combination of cryogenic electron microscopy (cryo-EM) with molecular dynamics (MD) simulations. Cryo-EM image intensity profiles across peptide-containing liposome membranes are analyzed by comparing them to synthetic images that are derived from MD trajectories of peptide-membrane systems representing different assumed binding modes. These simulated profiles serve as baseline models, which are then used to classify experimentally obtained images into respective categories. The approach was applied to influenza virus fusion peptides, providing evidence for predominantly transmembrane binding in pure POPC membranes and a transition toward surface-bound configurations upon the addition of cholesterol.

## 1 Introduction

Peptide-membrane interactions play a critical role in a number of biological functions, including membrane remodeling events such as poration,^1^ fusion^2^ or fission,^3^ signal transduction,^4^ or the attachment of larger protein structures via membrane anchoring fragments.^5^ A common characteristic of these processes is that they are initiated by specific interactions of just a few molecular moieties, which subsequently lead to mesoscopic-scale effects. As such, their comprehensive, mechanistic understanding requires microscopic insights into underlying molecular configurations. However, due to the small size, dynamic nature, and heterogeneous environment of membrane-bound peptides, determining their precise positioning within a lipid bilayer remains challenging for both experimental and computational modeling approaches.

A notable example is the influenza virus fusion peptide. It is a 23-residue-long N-terminal fragment of the HA2 subunit of viral hemagglutinin protein.^6^ During virus entry to the host cell,^7,8^ it anchors within the target membrane and initiates its merging with the viral lipid envelope.^9,10^ It has been intensively studied for decades as a model system for peptide-assisted fusion,^11,12^ but still, in spite of deciphering its presumably active conformation,^13^ the knowledge concerning its actual placement within the membrane and, hence, also detailed understanding of its fusogenic activity remain elusive.^14^ In its membrane-bound state, the peptide apparently forms a tight hairpin composed of two α-helical arms linked by a sharp kink.^15^ The resulting raft-like structure has hydrophobic and hydrophilic interfaces on its two opposite sides and distinct aromatic amino acids on its two poles. As the longer N-terminal helix has only 11 residues, it seems to be too short to be able to adopt any transmembrane orientation. Indeed, the analysis of indirect experimental measurements based on attenuated total reflection Fourier-transform infrared spectroscopy or spin-label electron paramagnetic resonance suggests that peptides insert only into the external membrane leaflet with the solvent-facing kink region and the N-helix tilted by 30–50° with respect to bilayer plane, thus partially burying the N-terminal group within the membrane core.^16−19^ Alternative studies based on nuclear magnetic resonance conclude that fusion peptides may actually orient parallel to the membrane surface, staying at the lipid–water interface with their hydrophobic face directed toward the membrane core and hydrophilic face exposed to the aqueous environment.^13,20^

Surface binding of fusion peptides, without disrupting membrane continuity, likely supports the classic fusion mechanism. This begins with the merging of two closely apposed proximal membrane leaflets to form a lipidic bridge called a stalk, followed by its expansion and the subsequent formation of an aqueous channel within the two remaining, initially distal, membrane leaflets.^21,22^ In such a mechanism, the peptide would assist in stalk nucleation, and the process would undergo without the possibility of content leakage from the two merging bodies into the environment. Such a nonleaky fusion mechanism was indeed confirmed by high-resolution cryo-electron tomography of HA-mediated fusion.^23^ At the same time, however, it was shown that some fusion events involve bilayer rupturing and the temporary existence of exposed membrane edges in direct contact with HA.^23,24^ This apparent leaky fusion phenotype was found to be associated with low cholesterol content in the target membrane. Notably, the occurrence of membrane discontinuity likely implies the ability of fusion peptides to adopt a membrane-spanning configuration that would facilitate bilayer rupture and eventually seal its exposed hydrophobic core. Such a possibility was experimentally confirmed by the observation of stable pores in POPC giant unilamellar vesicles whose formation was induced by the addition of fusion peptides.^25^ A support for transmembrane peptide configurations was also provided by a number of atomistic simulations.^26−30^ It was demonstrated that its stability can be maintained owing to peptide-induced membrane indentation, allowing to position the aromatic residues on hairpin poles within the lipid–water interface.^29^

The consequences of two alternative peptide binding modes in the context of membrane fusion were explored in a computational study, revealing two distinct mechanisms that could lead to the lowering of the associated free energy barrier.^31^ The first one, which assumes the surface-bound configuration, relies on the stabilization of early fusion intermediate by the rotation of the peptide hairpin from membrane-planar to membrane-perpendicular orientation and its fitting into the region of extreme negative curvature of membrane-water interface within the stalk. The second one, involving the transmembrane configuration, follows the so-called stalk-hole mechanism,^32^ in which fusion is facilitated by the relaxation of free energy cost needed to maintain a highly curved rim around membrane indentation caused by the peptide.

Despite its significance, the experimental evidence concerning the two possible binding modes of influenza virus fusion peptides remains inconclusive, primarily given the lack of clear agreement between the interpretation of different studies.^13,16−20,33^ In the current work, we propose a novel approach based on the combination of cryo-electron microscopy (cryo-EM) and molecular dynamics simulations to assess the possibility of surface and transmembrane positioning of the peptides within pure POPC and cholesterol-enriched membranes. In the light of experimental evidence indicating that peptide-induced membrane poration occurs even in the presence of cholesterol,^25^ it is feasible that some peptides permeate into the liposomes and bind to the inner membrane leaflet. Accordingly, we explicitly consider both external and internal surface binding scenarios. Although direct visualization of peptide units is not currently possible, the underlying assumption is that their presence should perturb the image intensity profile across the lipid bilayer in a specific manner, dependent on the actual binding mode. In analogy to massive averaging of 2-dimensional projections used to resolve 3-dimensional macromolecular structures, we accumulate and analyze 1-dimensional image intensity profiles across the lipid boundary to achieve the resolution suitable for the detection of the peptide presence. The reasoning about its position within the bilayer is based on using atomistic MD simulations of peptide-membrane systems to generate synthetic microscopic images representing each of the two considered binding modes. It provides us with baseline models that are used for a Bayesian-like inference to determine which of them favorably reflects the actual experimental data. Our findings provide support for primarily transmembrane peptide binding to pure POPC with a significant shift toward surface configurations upon the addition of cholesterol.

## 2 Methods

### 2.1 Experimental System

Large unilamellar vesicles were prepared by the extrusion methods. Lipids were purchased from Sigma-Aldrich and were dried from chloroform stock solution under a stream of nitrogen and subsequently under vacuum for 4 h to form a lipid film. The lipid film was then rehydrated with a buffer (10 mM citric acid/NaOH, 150 mM NaCl, pH 5.0) to achieve the final concentration of 2 mM lipid rehydration, which was done for 2 h with multiple vigorous vortexing steps. Eventually, the mixture went five times through a freeze–thaw cycle between the temperatures of liquid nitrogen and 55 °C, followed by extrusion (21 times) through 100 nm polycarbonate filters (Whatman) using Avanti Mini Extruder.

Fusion peptide (GLFGAIAGFIEGGWQGMVDGWYGSGKKKKD) was custom ordered with purity >95% (Lipopharm, Gdansk, Poland). Its N-terminus was unmodified, and the C-terminus was an amide. The -SGKKKD sequence was introduced to increase peptide solubility.^27,34^ Stocks were prepared from weighted amounts dissolved in water as 300–500 mM solutions. Concentrations were checked by UV spectroscopy using the extinction coefficient at 280 nm of 12490 M^–1^ cm^–1^.

For cryo-EM imaging of peptide-containing systems, an aliquot of 2 mM liposomes was incubated with 40 μM peptide at 40 °C for 3 min. Subsequently, 3 μL of liposome or liposome-peptide solution was applied onto a glow-discharged continuous carbon-coated TEM grid, blotted, and fast frozen at 22 °C and 100% relative humidity in an FEI Vitrobot Mark IV (FEI), followed by 1 min break and repeated application of 3 μL solution. The sample was blotted and fast frozen at 22 °C and 100% relative humidity.

### 2.2 Experimental Image Processing

**Figure 1:**
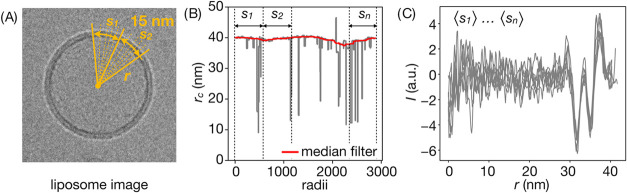
Microscope image processing. (A) Liposome image with individual radial profiles grouped into segments. (B) Determined location of the bilayer center for all radial profiles smoothed with median filter. (C) Intensity profiles averaged within subsequent segments in arbitrary units (au).

Images were taken using 200 kV cryo-Transmission Electron Microscope Glacios under 3 μm defocus, as 4096 × 4096 bitmaps with pixel size corresponding to 0.1586 nm. To obtain radial profiles of image intensity, we implemented the following procedure. (1) An image region containing an isolated (not overlapping with others) liposome with several nm margins was manually selected using circular selection in the ImageJ program.^35^ (2) Pixel gray levels in 2880 evenly distributed directions originating from the center of the circle were collected with 0.1 nm intervals in radial coordinates (Figure 1A). (3) For each such profile, a likely location of intensity bands corresponding to the lipid bilayer was determined by finding the maximum of its convolution with a kernel function representing a model transmembrane intensity profile. For each of the four considered systems (two lipid compositions, with and without peptides), during the first preliminary pass, we used a kernel being a combination of three Gaussian functions, and for the second final pass, we reprocessed all data with a kernel determined based on an average profile obtained based on the first pass. (4) A baseline location of bilayer intensity around the entire liposome was calculated using a median filter encompassing 301 nearest profiles – this served to filter out poorly resolved profiles with ambiguous location of intensity peaks (Figure 1B). (5) Individual profiles were partitioned into sectors corresponding to ∼15 nm arc length on the liposome circumference. (6) An average profile was then calculated for each sector in which at least 95% of individual radial profiles had an estimated location of bilayer center within 0.5 nm from the baseline median value (Figure 1C).

In order to standardize the amplitudes of the obtained intensity profiles, we performed normalization with respect to mean value and standard deviation of the gray level ensemble, {*I*_o_}, such that for each profile, it was based on the region that corresponded to bulk ice on the outer side of liposome area, i.e., extended between 6 and 9 nm from the central peak (Figure 3A,B). To check whether such obtained sets of processed profiles for peptide-free and peptide-containing systems share uniform levels of standardized intensity, *Ĩ*_o_, we compared the distributions of scaled intensity *p*(*Ĩ*_o_), using nonparametric, 2 sample Kolomogorov-Smirnov test. The resulting distributions are provided in Supporting Information (SI). The number of processed liposomes and resulting averaged profiles for each system are given in Table 1.

**Table 1:** Experimental Data: Number of Analyzed Liposomes, Initial Intensity Profiles, and Profiles Selected for Analysis Using z-Score Filter

system	liposomes	all profiles	selected profiles (*N*)
POPC	115	699	298
POPC+P	227	1346	683
CHL30	42	282	113
CHL30+P	32	195	111

### 2.3 Molecular Dynamics Simulations

Considered systems included atomistically modeled planar lipid bilayers composed of POPC lipids or POPC with 30% cholesterol (CHL30), embedded in an explicit aqueous environment with 0.15 mol/L NaCl, and simulated under periodic boundary conditions. HAfp were modeled as 23-amino acid-long sequences, GLFGAIAGFIEGGWQGMVDGWYG, in helical hairpin conformations with charged N-terminus and amidated C-terminus, and initial geometry adopted from the NMR structure (pdb 2kxa, model 1).^13^ Equilibrated, transmembrane, or surface-bound configurations were taken from our previous study.^29^ A list of particular system sizes, including peptide-to-lipid ratios (lipids in both membrane leaflets are counted) is provided in the SI. Peptides and lipids were modeled with Amber99SB-ILDNP*^36^ and Amber Lipid14^37,38^ force fields, respectively, and TIP3P water model.^39^ Covalent bonds with hydrogen atoms were constrained using LINCS algorithm,^40^ we used 1.0 nm cutoff for van der Waals interactions, and electrostatic interactions were treated using the particle mesh Ewald method^41^ with 0.12 nm mesh spacing and 1.0 nm cutoff in real space. Simulations were propagated using a 2 fs time step, at a temperature of 310 K, and a pressure of 1 bar, maintained by velocity-rescale thermostat and Parrinello–Rahman^42^ barostat, respectively. All MD runs were carried out using Gromacs software.^43^ All synthetic intensity profiles were generated independently using 1 μs simulation blocks. The systems including surface-bound peptides served to construct intensity profiles representing both external and internal surface binding modes by simply inverting the underlying transmembrane phase-shift profiles along radial coordinates. A detailed list of simulated systems, simulation times, and the number of considered blocks for each system is provided in the SI.

### 2.4 Microscopic Image Construction

The procedure used to construct simulated cryo-EM images was based on weak phase approximation, following the approach proposed in refs (44) and (45). Briefly, image contrast was assumed to arise due to the phase shift in the electron wave function and its interaction with electrostatic potential variations along the way. The underlying effect was described by a position-dependent phase-shift profile, γ(*w*), which includes contributions from neutral atoms and from the surrounding electrostatic potential, ϕ(*w*). The former were estimated from the atomic number densities, ρ_*i*_(*w*), and atom-specific, shielded coulomb potentials, *V*_i_, such that
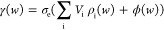
1where σ_e_ describes the first-order dependence of electron phase shift on the projected potential and was taken as 7.3 mrad V^–1^nm^–1^ for 200 keV microscope.^44^ The values of *V*_i_ coefficients were taken from the literature as 25, 130, 108, 97, and 267 VÅ^3^ for H, C, N, O, and P, atoms, respectively.^46^

**Figure 2:**
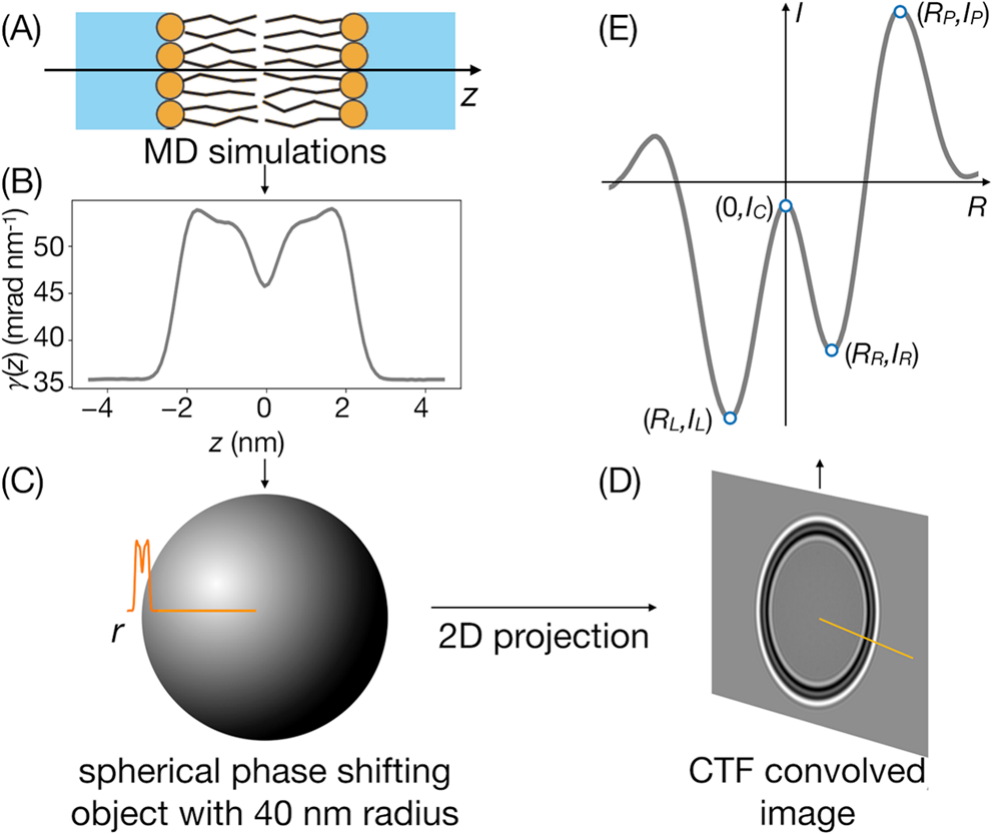
Scheme of microscopic image construction. (A) Simulated system with planar bilayer (for clarity, no peptide is shown). (B) Phase-shift profile along bilayer normal. (C) Construction of spherical phase-shifting object. (D) Phase-shift projection on a 2D plane and convolution with contrast transfer function (CTF). (E) Radial intensity profile across bilayer with abbreviations of the profile descriptors used.

Initially, the phase-shift profile was obtained along the normal, *z*, of a planar lipid bilayer embedded in an aqueous environment with or without peptides bound in a specific configuration (Figure 2A,B). Atomic number densities were explicitly calculated based on atomistic MD trajectories. The external electrostatic potential profile was calculated by double integration of charge density, ρ_c_(*z*):
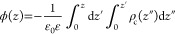
2where *ε*_0_*ε* are the vacuum and relative electric permittivity, respectively, with the latter assumed to be 1 due to the use of a fully atomistic simulation model. Having obtained γ(*z*), we used it to construct a 3-dimensional distribution of the phase-shift function for a model spherical liposome of *r*_0_ radius surrounded by aqueous solution: γ(**r**) = γ(|**r**|− *r*_0_). We used *r*_0_ = 40 nm for POPC systems and *r*_0_ = 50 nm for CHL30 systems to match the average radii from the corresponding experimental ensembles (see SI Figure 1for radii distributions). Subsequently, we numerically calculated the projection of γ(*r*) function on a 2-dimensional plane, obtaining radial phase-shift distribution γ(*r*) (Figure 2C,D).

Image intensity, *I*(*R*), was determined by the convolution of γ(*r*) with contrast transfer function (CTF). This was performed in reciprocal space as
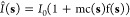
3where **s** is the spatial frequency, m is the scaling factor, *Î*(**s**) and f(**s**) are the Fourier transforms of *I*(*r*) and γ(*r*), respectively, and c(**s**) is the CTF, defined as
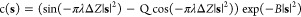
4Here, λ = 2.51·10^–3^ nm is the electron wavelength for a 200 keV microscope, Δ*Z* is the defocus length, Q is the amplitude contrast factor, and *B* is the amplitude decay coefficient. The values of Δ*Z* = 2.4 μm, Q = 0.04, and *B* = 2 nm^2^ were determined by fitting the positions and amplitudes of calculated extrema in the intensity profile for pure POPC membrane to those in the average experimental profile for respective liposome type. The fit was performed by minimizing the root-mean-square difference of peak positions, with weights for radial and amplitude coordinates inversely proportional to their standard deviations in experimental profiles. The final image intensity in real space was recovered using the inverse Fourier transform as .

### 2.5 Distribution of Profile Descriptors

Radial and amplitude coordinates of the extrema in experimentally determined intensity profiles for peptide-free and peptide-containing systems (Figure 2E) were used to construct sets of 7-dimensional profile descriptors {**p** = (*R*_L_, *R*_R_, *R*_P_, *I*_L_, *I*_C_, *I*_R_, *I*_P_)}. For analysis, we included only profiles for which all coordinates exhibited a z-score <1.5 based on their initial underlying distributions (Table 1, selected profiles). Such selected ensembles of profile descriptors were standard scaled according to averages and standard deviations determined based on peptide-free systems separately for the analysis of POPC and CHL30 liposomes. Similar, 7-dimensional descriptors were calculated for simulated intensity profiles, and the expected shifts in their positions in response to increasing peptide concentrations, *C*_P_, under the surface and transmembrane binding scenarios served to determine sets of reference points {**u**(*C*_P_)}. The positions of the reference points were uniformly shifted such that **u**(0) corresponded to the location of the maximum density in {**p**} distribution for the respective experimental peptide-free system and scaled according to the same scaling factors as the ones used for experimental profiles.

The fraction of the experimental ensemble assigned to a particular reference point **u**(*C*_P_) was based on determining the number of intensity profiles, *n*, whose corresponding points, **p**, in the 7-dimensional descriptor space were within the Cartesian distance threshold, *T*, from **u**(*C*_P_) and dividing it by the total number of intensity profiles of a given kind, *N*:
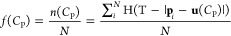
5where H is the Heaviside step function. The threshold was adjusted in such a way that *f*(0) = 0.9 for the peptide-free ensemble. Statistical significance of the excess of ensemble fraction representing peptide-free or peptide-containing systems assigned to any reference point was evaluated using the *χ*^2^ test.

Subsequently, the reference points for which such statistically significant difference was observed were partitioned into five categories: representative of unbound membrane, if significantly more peptide-free profiles were assigned to them, or, if significantly more peptide-containing profiles were assigned to them, representative of surface or transmembrane peptide binding, and depending on their source model. The remaining reference points were deemed inconclusive.

Finally, to estimate the fraction of experimental intensity profiles representing peptide-free membrane or any of the proposed peptide binding modes, we partitioned all profiles to their nearest reference points **u**_*C*_P__ and assigned to a unique category (unbound, external or internal surface-bound peptide, transmembrane peptide, undefined) based on the previously determined type of these points.

## 3 Results and Discussion

### 3.1 Experimental Intensity Profiles

**Figure 3:**
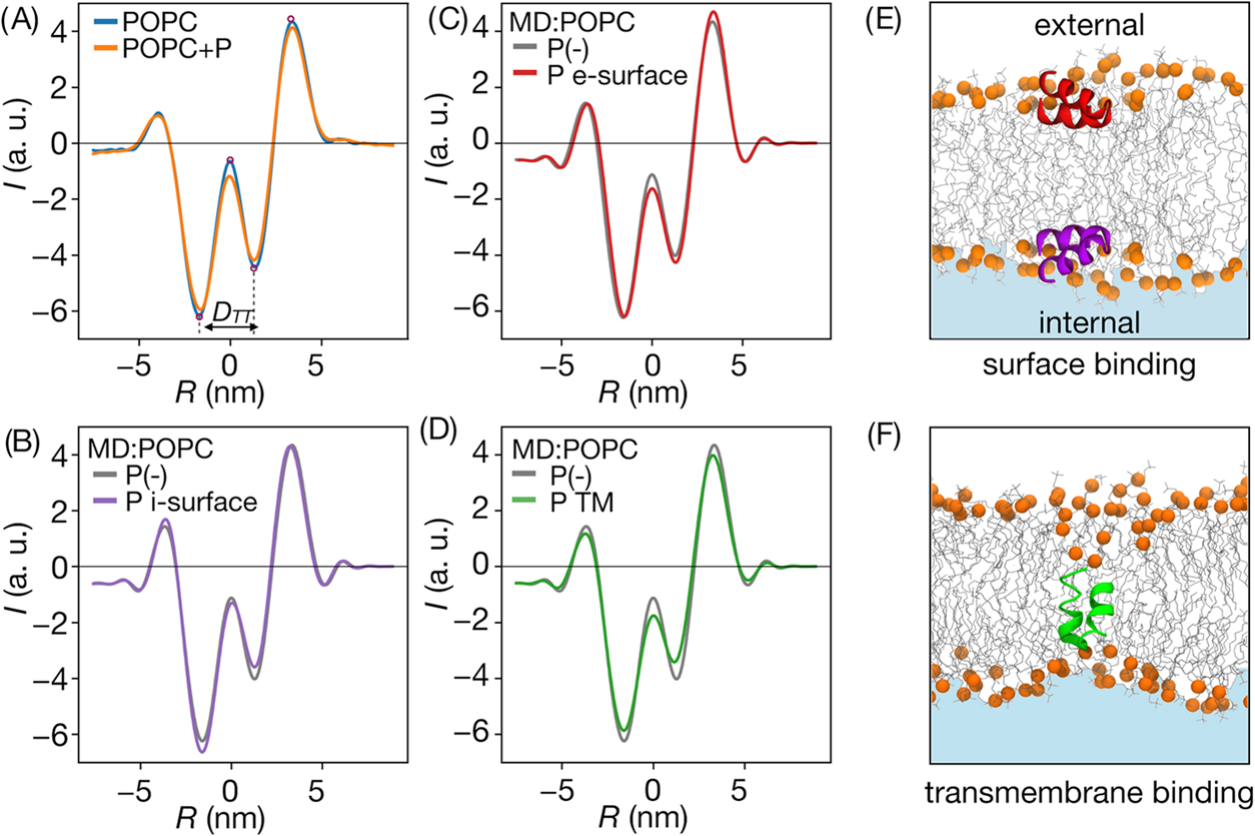
Experimental and simulated intensity profiles for POPC liposomes. (A) Experimental profiles for peptide-free and peptide-containing liposomes. (B–D) Simulated profiles for pure POPC membrane and three peptide binding modes at P/L 3.1 × 10^–2^ for internal and external liposome surface bindings and 1.5 × 10^–2^ for TM mode. (E and F) Simulation snapshots from single peptide simulations (P/L 0.62 × 10^–2^) illustrating respective peptide binding modes (note: panel E represents the artificially mirrored membrane, just for illustration purposes).

Intensity profiles across the lipid bilayer reveal five prominent extrema (Figure 3). Two local minima correspond to two dark bands in the microscopic image of a liposome wall. They arise due to electron scattering on lipid headgroup regions, with primary contribution from their phosphorus atoms. The minima are separated by a maximum that is, however, still somewhat darker than the background. The entire low-intensity region is flanked by two intensity maxima (light bands), of which the one corresponding to the outer side of the liposome structure is significantly more pronounced.

The observed distance between intensity minima, known as the trough-to-trough distance, *D*_TT_,^45^ is found to be 3.11 ± 0.04 nm in the case of pure POPC liposomes (Figure 3). It is notably smaller than the widely known headgroup-to-headgroup distance, *D*_HH_, defined based on maxima of electron densities colocated with phosphorus atoms and reaching 3.9 nm for POPC bilayers.^47^ This discrepancy arises from the fact that, contrary to its appearance, the microscopic image of a liposome wall does not represent a direct cross section through the lipid bilayer. It should rather be regarded as a 2-dimensional projection of a spherical phospholipid boundary, whose details are further affected by the properties of CTF. Nonetheless, *D*_TT_ has been shown to be a monotonic function of *D*_HH_,^45^ and hence, its changes directly reflect variations in membrane morphology. Given this notion, the presence of fusion peptides apparently leads to a slight narrowing of bilayer thickness, as an average *D*_TT_ measured for POPC+P liposomes was 3.01 ± 0.03 nm.

Unfortunately, further reasoning about the effects of peptide presence based on other peak-to-peak distances along radial coordinates is hampered by their limited interpretability and the fact that the differences between the two systems are only at the border of statistical significance. Accordingly, to gain meaningful insights into peptide-related modifications of intensity profiles, we considered the amplitudes of the observed peaks. The analysis here is complicated by the fact that raw gray levels of liposome images that contribute to the final intensity peaks depend on the particular setup of the microscope system as well as on the protocol of subsequent image processing. In order to mitigate this issue, we normalized gray levels contributing to each individual intensity profile (see Methods for details) and verified that their resulting distributions for peptide-free and peptide-containing systems were statistically indistinguishable (*p* ∼ 0.15 in a two-sample Komologorov-Smirnof test, SI Figure 4B).

The analysis of such normalized intensity profiles reveals general dampening of intensity oscillations due to peptide presence: the depths of both minima as well as the amplitude of the right peak are smaller in peptide-containing systems, and the central peak is less pronounced (Table 2).

**Table 2:** Major Peak Positions in Experimental and Calculated Intensity Profiles^a^

system	positions (nm)		
	*R*_L_	*R*_R_	*R*_P_
POPC	–1.73 ± 0.03	1.38 ± 0.02	3.41 ± 0.03
POPC+P	–1.70 ± 0.02	1.31 ± 0.02	3.40 ± 0.03
MD:POPC	–1.61	1.28	3.34
CHL30	–1.87 ± 0.03	1.50 ± 0.04	3.67 ± 0.04
CHL30+P	–1.79 ± 0.04	1.39 ± 0.05	3.59 ± 0.05
MD:CHL30	–1.78	1.40	3.42

	intensities (au)			
	*I*_L_	*I*_C_	*I*_R_	*I*_P_
POPC	–6.08 ± 0.19	–0.57 ± 0.09	–4.38 ± 0.13	4.96 ± 0.15
POPC+P	–5.87 ± 0.21	–0.94 ± 0.08	–4.13 ± 0.09	4.66 ± 0.11
MD:POPC	–6.56	–1.19	–4.23	4.56
CHL30	–6.88 ± 0.25	–0.90 ± 0.17	–4.23 ± 0.21	5.88 ± 0.30
CHL30+P	–6.55 ± 0.21	–1.46 ± 0.23	–3.96 ± 0.24	5.85 ± 0.30
MD:CHL30	–7.25	–0.37	–4.93	4.91

a Radial coordinates: *R*_L_ – left minimum, *R*_R_ – right minimum, *R*_P_ – right peak. Intensity amplitudes: *I*_L_ – left minimum, *I*_R_ – right minimum, *I*_R_ – right peak, and *I*_P_ – central peak. See Figure 2E for illustration. Radial coordinates are shifted such that *R* = 0 for the central intensity maximum.

### 3.2 Calculated Intensity Profiles

Intensity profiles constructed based on MD simulations of lipid membranes reproduce the arrangement of all major, experimentally resolved extrema along the radial coordinate (Figure 3B–D). Close inspection of plots obtained for pure POPC system reveals, however, that the positions of all peaks are shifted by ∼0.1 nm toward the central maximum compared to those established based on microscopic data (Table 2). It results in the calculated trough-to-trough distance, *cD*_TT_ = 2.89 nm that is shorter by ∼0.2 nm than the experimental one, even though the headgroup-to-headgroup thickness of simulated POPC bilayer agrees well with the literature value reported for this kind of membrane.^48^ The amplitudes of simulated intensity peaks also qualitatively follow their experimental counterparts; however, the depths of left and right minima are slightly over- and underestimated, respectively.

The observed quantitative discrepancies are likely attributable to the fact that the construction of simulated profiles does not take into account the asymmetry of lipid packing within curved liposome walls. In addition, the model assumes the perfect spherical shape of lipid vesicles, thus neglecting fluctuations that likely contribute to the smearing of the intensity profiles resolved based on microscopic images. It is important to note, however, that such effects are expected to similarly impact the simulated profiles of both pure lipid and peptide-containing systems, and thereby, the differences between them should replicate the trends observed based on real images.

To this end, the modifications of intensity profiles introduced by the presence of peptides are subtle. Most notable are differences in radial and amplitude coordinates corresponding to TM and external surface binding modes (Figure 3C,D). The inclusion of externally bound peptide is found to slightly increase the depth of the right minimum and the height of the right peak compared to the peptide-free system, whereas the TM peptide placement leads to an opposite effect and an additional decrease in the amplitude of the left minimum. In turn, the internal binding mode results in intermediate changes within the right-hand side of the intensity profile but leads to a distinct deepening of the left minimum (Figure 3B).

### 3.3 Effects of Peptide Concentration

The comparison of experimental and simulated profiles is complicated by the lack of information concerning the details of peptide binding to liposomes under experimental conditions. This encompasses uncertainty related to an average peptide-to-lipid ratio (P/L) but also to the nature of their binding modes and the variability of their distribution on liposome surfaces. Consequently, intensity profiles obtained for the POPC+P system can represent a heterogeneous mixture of membrane patches including deeply and surface-bound peptides, with concentration spanning from peptide-free regions to those potentially decorated with dense peptide aggregates. Given this notion, the interpretation of differences between average intensity profiles for POPC and POPC+P systems in light of simulated data that would reflect any single, arbitrary chosen conditions can be insufficient.

**Figure 4:**
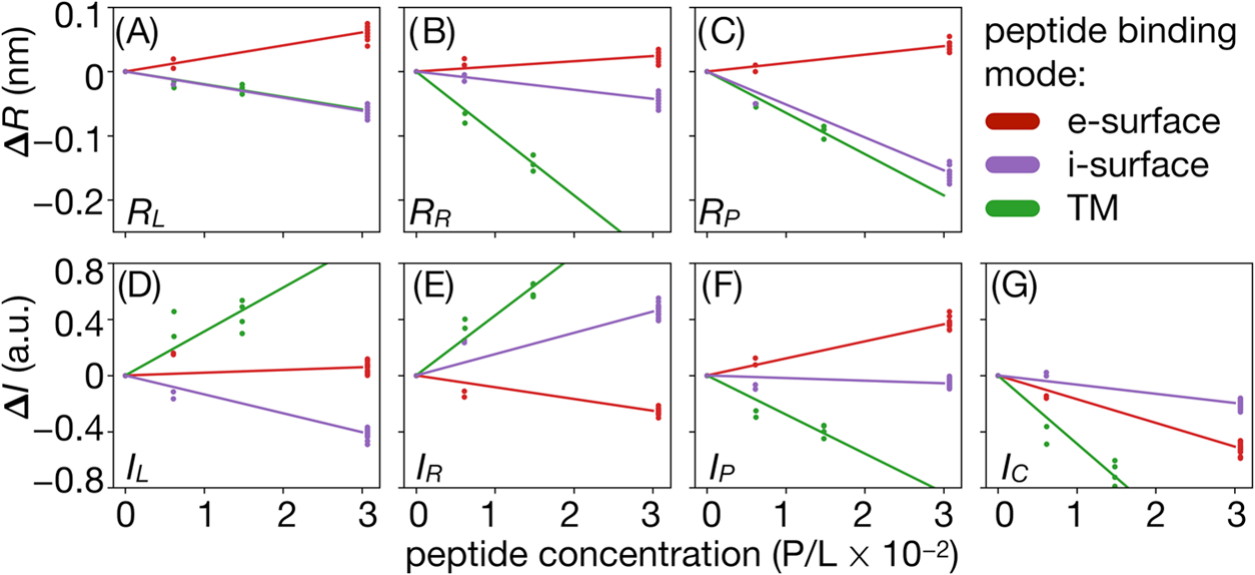
Changes in peak positions for increasing peptide concentrations; points: simulation data obtained based on independent trajectory blocks; lines: linear fits.

To investigate how simulated intensity profiles respond to changes in peptide concentrations, we constructed a series of variants based on available MD trajectories carried out at different P/L both for surface and TM peptide placements (SI, Table 1). The analysis of peak positions along radial and amplitude coordinates as peptide concentration increases reveals clear linear-like trends (Figure 4A–G), even though some peptide aggregation is observed at higher P/L (see SI Section 7 for aggregation evidence). At present, it remains unclear to what extent the aggregation would affect the concentration dependence of intensity profiles, particularly in the case of surface-bound peptides. While peptide assemblies observed in our simulations appear to remain on the membrane surface (SI Section 7), it was demonstrated that under higher peptide-to-lipid ratios, aggregates may eventually displace lipids within the *cis* membrane leaflet.^25^ The occurrence of such events can be somewhat suppressed by the use of periodic boundary conditions, and they may become more likely for larger systems even at peptide concentrations considered in our model. Still, unless extreme aggregation is the dominant and only peptide binding mode on the membrane surface, it appears that a simple linear function of average peptide concentration provides a reasonable, first-order approximation of the underlying trends. Such a model enables the construction of simulated intensity profiles corresponding to desired P/L through linear interpolation or extrapolation of existing data. A subsequent assessment of conditions providing their best agreement with the *distribution* of experimentally resolved intensity profiles for the POPC+P system gives the possibility of Bayesian-like estimation of the most likely model of peptide binding.

### 3.4 Distribution of Profile Descriptors

**Figure 5:**
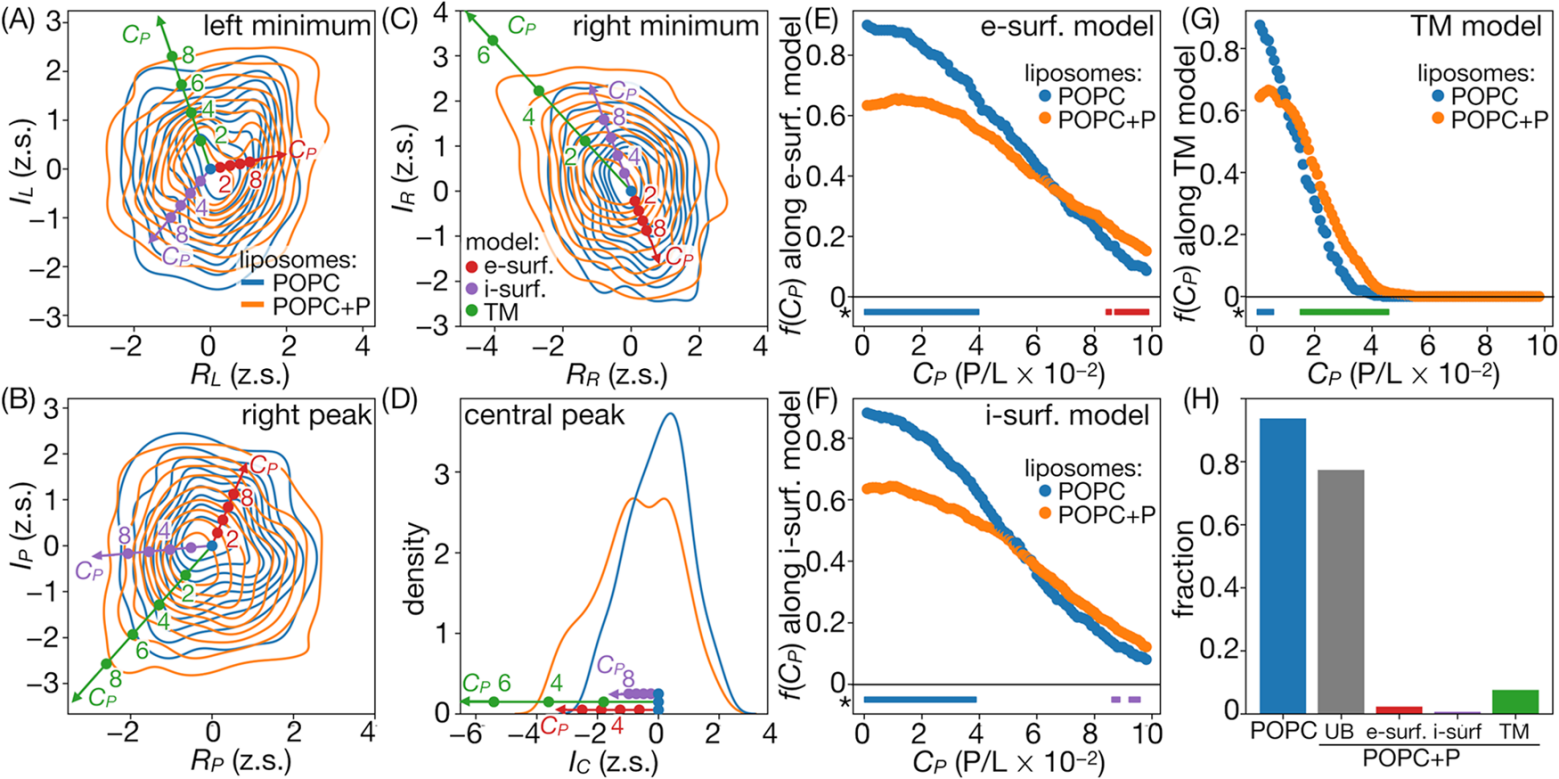
Distribution of peak descriptors for POPC systems. (A–D) Projections on radial and amplitude coordinates with reference points illustrating simulation-based trends for increasing peptide concentrations. (E–G) Fractions of experimental distributions, *f*(*C*_P_), assigned to respective reference conditions. Points under plots represent *C*_P_ ranges with statistically significant differences in *f*(*C*_P_) for peptide-free and peptide-containing systems. (H) Fractions of intensity profiles attributable to specific peptide binding modes, with UB denoting profiles obtained for peptide-containing systems that are not indicative of binding.

The positions of major peaks and minima within the intensity profiles can be described by a set of 3 positions along radial coordinates and 4 amplitudes, together forming a 7-dimensional descriptor (Figure 2E). The comparison of normalized probability density distributions for these descriptors obtained based on individual profiles gathered for POPC and POPC+P is shown in the form of 2-dimensional projections in Figure 5A–D. Its analysis corroborates the general trends discussed above in regard to averaged intensity profiles but also reveals considerable dispersion and a large degree of overlap between the two systems. To some extent, it is the expected result of inherently noisy cryo-EM data; however, notably, broader distributions for the POPC+P system may reflect additional heterogeneity of membrane patches related to peptide binding. The latter possibility might be further supported by the multimodal nature of the central peak intensity, *I*_c_, which is apparent in the case of the peptide-containing system and absent in the peptide-free system (Figure 5D).

The projection of modeled descriptor values for increasing peptide concentrations, *C*_P_, indicates clearly opposing trends in the positions of the right intensity minimum and the right peak between the external surface and TM peptide binding models. In turn, the left minimum offers less discriminatory power between these two models but shows the distinct response to the internal surface binding. In general, peptide binding in TM configuration appears to cause larger deviations in the structure of the intensity profiles than surface binding at the same *C*_P_, which seems reasonable given the obvious difference in the depth of bilayer penetration and the expected degree of its perturbation.

### 3.5 Peptide Binding Mode

To determine peptide binding mode and concentration that best explain the differences in the distribution of descriptors derived from experimental intensity profiles for POPC and POPC-P systems, we adopted a Bayesian-like approach. We first defined a set of reference points, {**u**(*C*_P_)}, in the 7-dimensional descriptor space, representing either of the two surface binding modes or the TM binding mode at increasing *C*_P_. For each such reference point, we then calculated the fractions of the experimental ensembles for POPC and POPC-P systems that fell within a specified cutoff radius. The radius was chosen such that a hypersphere corresponding to *C*_P_ = 0 contained 90% of the data points from the pure POPC system. The resulting fractions of POPC and POPC-P data as a function of peptide concentrations, *f*(*C*_P_), obtained for all considered binding modes are shown in Figure 5E–G.

Despite the substantial overlap between descriptor distributions for POPC and POPC-P systems, *f*(*C*_P_ = 0) is significantly higher (*p* < 0.05) for experimental data based on pure liposomes in comparison to those mixed with peptides. Nevertheless, a considerable fraction of the intensity profiles for the peptide-containing system (∼70%) is not clearly distinguishable from 90% of the most typical profiles from the peptide-free system. Possibly, it indicates that the peptide distribution on the liposome surface may be uneven, including also regions of unaffected membrane.

As *C*_P_ is increased to 2–4 peptides per 100 lipids, the reference intensity profiles modeled according to the TM binding model but not to any of the surface binding models become representative of a significantly higher fraction of the experimental POPC-P ensemble compared to the POPC ensemble. In the case of reference points corresponding to both surface models, such a condition is reached only at considerably higher *C*_P_ that reaches 8–10 peptides per 100 lipids. Given that the simulated intensity profiles at this P/L range appear to be representative only for a small fraction of experimental ensembles, the above analysis seems to generally favor the TM binding model of peptides to POPC liposomes (Figure 5H).

### 3.6 Effect of Cholesterol

The general morphology of the intensity profiles obtained for CHL30 liposomes is the same as in the case of pure POPC vesicles, with only minor differences in positions and relative amplitudes of the peaks (SI Figure 4C, Table 2). The addition of cholesterol is expected to increase bilayer thickness, and indeed, this effect is reflected by the obtained *D*_*TT*_ = 0.37 ± 0.05 nm, which is by ∼0.26 nm larger than the one described above for the POPC membrane. The corresponding difference determined for calculated profiles was found to be 0.29 nm, which aligns well with the experimental findings. This correspondence supports the validity of our simulation-based model for the assessment of relative changes in the intensity profiles, even though the absolute positions of the peaks with respect to the central maximum are systematically underestimated.

**Figure 6:**
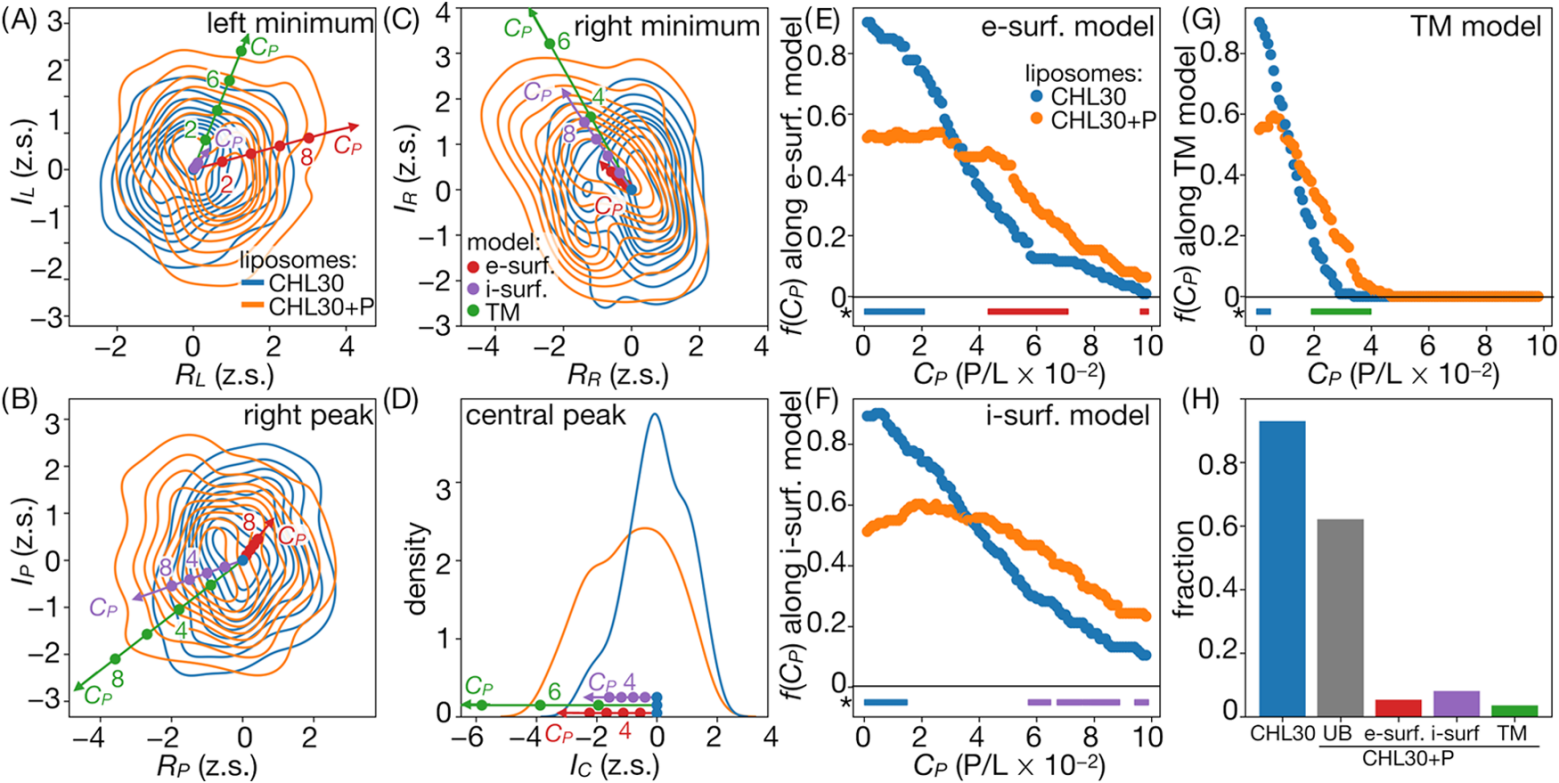
Distribution of peak descriptors for CHL30 systems. (A–D) Projections on radial and amplitude coordinates with reference points illustrating simulation-based trends for increasing peptide concentrations. (E–G) Fractions of experimental distributions, *f*(*C*_P_), assigned to respective reference conditions. Points under plots represent regions with statistically significant differences in *f*(*C*_P_) for peptide-free and peptide-containing systems. (H) Fractions of intensity profiles attributable to specific peptide binding modes, with UB denoting profiles obtained for peptide-containing systems that are not indicative of binding.

Remarkably, also an examination of changes in peak positions that are predicted to occur as an effect of increasing P/L (Figure 6A–D, SI Figure 6) indicates qualitatively the same relative trends for different peptide binding modes as in the case of pure POPC system (Figure 4). Unfortunately, however, their direct mapping on experimental descriptor distributions suggests possibly inferior discriminatory power between TM and surface peptide binding models compared to the case of pure POPC liposomes. Clearly, diverging behavior is seen only in the case of shifts resulting from external surface binding within the right peak radial and amplitude coordinates (*R*_P_, *I*_P_), whereas the remaining descriptors reveal trends that are, to a large degree, colinear. Nonetheless, the analysis of a fraction of experimental data that is best explained by the underlying models indicates *C*_P_ ranges with clear enrichment of supposedly peptide-containing profiles for both TM and surface binding scenarios. Interestingly, in comparison to systems containing POPC liposomes, the profiles consistent with surface-based models occur at lower *C*_P_ (∼4 to 9 peptides per 100 lipids) and, thus, now constitute a considerable fraction of all obtained cases (Figure 6H).

### 3.7 Classification of Intensity Profiles

**Figure 7:**
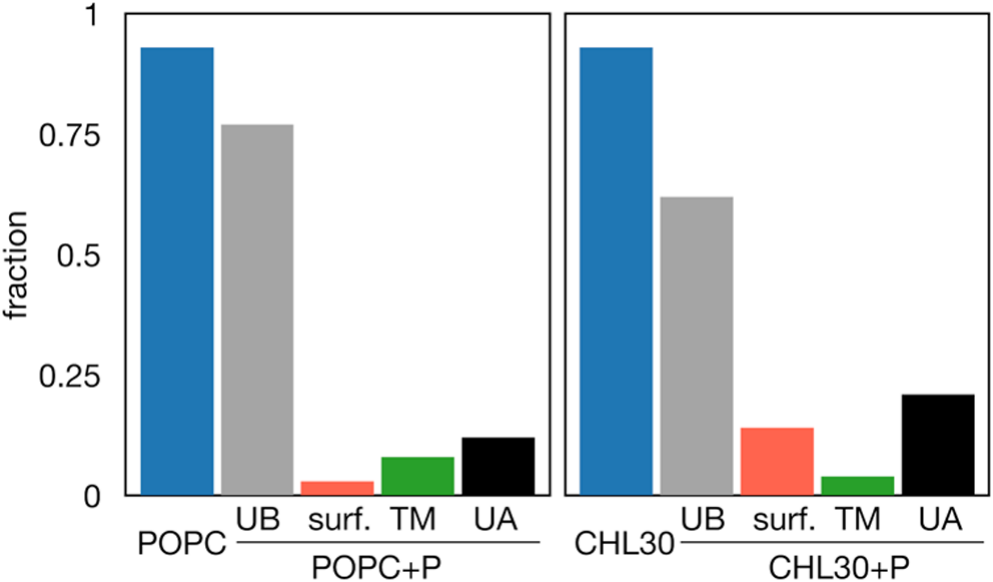
Total fractions of intensity profiles attributable to specific peptide binding modes in POPC and CHL30 systems. UB – profiles not indicative of binding, UA – profiles for peptide-containing systems that remained unassigned to any class.

To estimate the general distribution of intensity profiles representing the considered models of peptide binding, we partitioned the experimental data into distinct states based on the predominant fraction of experimental ensembles assigned to specific reference points within the 7-dimensional descriptor space (Figure 7, see Methods for details). Taking into account the entire range of considered P/L, we find that 77 and 62% of intensity profiles for peptide-containing POPC and CHL30 systems, respectively, can be assigned to reference points which gather significantly larger fraction of profiles associated with corresponding peptide-free systems. Consequently, these profiles should not be interpreted as indicative of peptide binding. To some extent, this might reflect noise-related uncertainty in experimental data, but the possibility of uneven peptide distribution across the liposome surface, resulting in peptide-free membrane patches, cannot be excluded either.

In the case of the POPC system, the fraction of profiles classified as consistent with TM peptide configuration was significantly larger than the one corresponding to external or internal surface binding mode (8 vs 3%). In contrast, for the CHL30 system, these fractions were 4 and 14%, respectively, implying a relative depletion of TM configurations and enhancement of surface binding mode in the presence of cholesterol. This finding supports the notion that relatively short hairpin conformation is less likely to adopt a transmembrane configuration in a thicker bilayer. Remarkably, this is in qualitative agreement with experimental evidence indicating that liposome leakage induced by influenza virus fusion peptides decreases from 75 to 50% upon transition from POPC to 70:30 POPC/cholesterol membrane composition.^25^ A direct, quantitative comparison is hampered, however, by uncertainties arising due to likely heterogeneous distribution and architecture-related permeation efficiency of membrane pores formed by the peptides.

Finally, we note that non-negligible fractions of intensity profiles for peptide-containing systems, that is, 14 and 21% for POPC and CHL30 liposomes, respectively, were not associated with any of the proposed simulation models (Figure 7, UA fraction). Aside from outliers with randomly accumulated artifacts resulting from experimental image generation and processing, they might include profiles that reflect complex peptide arrangements not explicitly considered in simulations. The first obvious candidate would be membrane patches with peptides bound on both internal and external leaflets. Once peptide permeation into the liposomes is envisioned, such configurations are expected; however, their explicit simulations accounting for independent concentration on each bilayer leaflet would be challenging. A crude approximation of the likelihood of their occurrence would be the product of probabilities reflecting external and internal binding that would give ∼0.02 and ∼0.5% for POPC and CHL30 systems, respectively. Another possibility, likely reflecting a higher unassigned fraction in CHL30 systems, might be related to peptide-induced membrane phase separation. The propensity for such phenomenon in peptide-treated POPC-cholesterol giant unilamellar liposomes was indeed observed in our earlier study,^27^ and it was not accounted for in the current simulation model.

## 4 Conclusions

In this work, we employed a novel, probe-free approach that combines cryo-EM imaging and molecular dynamics simulations to determine the positioning of membrane-bound peptides. We gathered a series of cryo-EM images of free liposomes and liposomes treated with influenza virus fusion peptides and collected the respective, averaged image intensity profiles across liposome walls. Subsequently, we calculated theoretical intensity profiles based on atomistically simulated lipid bilayer systems in peptide-free scenarios or with peptides bound in either surface or transmembrane configuration. The obtained synthetic images provided insights into the changes in the morphology of intensity profiles expected for different ways of peptide positioning, thus allowing the interpretation of original experimental data in terms of the most likely binding mode.

Given inherently noisy cryo-EM images, the uncertainties of the introduced descriptors based on positions and amplitudes of intensity peaks turned out to be relatively low, reaching sub 0.05 nm for positional values and less than one-third of an arbitrary unit that corresponded to the standard deviation of background image intensity fluctuations. Still, however, the changes of individual peak attributes between averaged experimental intensity profiles corresponding to pure and peptide-containing membranes were subtle and remained at the border of statistical significance. For the sake of quantitative analysis, this issue was mitigated by considering joint variability of peak positions and amplitudes in a form of a 7-dimensional descriptor.

Another challenge for obtaining quantitative results arose from the likely uneven distribution of peptides on liposome surface, which precluded making arbitrary assumptions concerning P/L in simulation systems used to obtain baseline models. However, the changes in the considered attributes of theoretically obtained intensity profiles were found to depend linearly on peptide concentrations both in the case of surface and transmembrane binding scenarios. This linearity allowed interpolation between explicitly considered simulation conditions and extrapolation to higher peptide concentrations. It is important to note that the validity of this later procedure may be limited due to the likely aggregation of the peptides at high concentrations such as P/L ∼0.1, which can qualitatively alter membrane organization, rendering current simulation-based models unsuitable. Indeed, such a possibility manifested by local, complete displacement of lipids from one membrane leaflet by peptide aggregates was suggested in a study by Rice et al.^25^ as a putative fusogenic mechanism of influenza virus fusion peptides. Intriguingly, even under the current linearity assumption, our results indicate that surface peptide binding occurs only at high P/L, which may be consistent with their tendency to aggregate.

On the contrary, at moderate P/L, we find evidence for TM peptide binding, especially in pure POPC systems. Although such a binding mode for relatively short hairpins formed by influenza virus fusion peptides has been traditionally dismissed, recent independent simulation studies, indirect experimental measurements, as well as recent observations of membrane poration in response to peptide binding suggest its plausibility. It is reasonable to assume that membrane-spanning configurations become less likely in thicker, cholesterol-containing bilayers. While our current results still indicate such a possibility in CHL30 systems, it remains an open question whether it plays a role in physiological conditions in which the virus fuses with the cholesterol-rich endosomal membrane.

## Supporting Information Available

Statistics of liposome radii, list of simulated systems, detailed decomposition of the phase-shift profile, data on amplitude scaling, intensity profiles for cholesterol-containing systems, dependence of descriptor values on peptide concentration in cholesterol-containing systems, analysis of peptides positions within the membrane, and aggregation during MD simulations (PDF)
