# Supplementary material for: Integrating Cryo-Electron Microscopy and Molecular Dynamics Simulations to Investigate Membrane Binding of Influenza Virus Fusion Peptides
Source: J Am Chem Soc. 2025 Apr 11;147(16):13385–95. doi: 10.1021/jacs.4c18441 (PMC12023020; doi:10.1021/jacs.4c18441)
Supplement: Supplementary file 1 — ja4c18441_si_001.pdf [file ja4c18441_si_001.pdf]

Integrating Cryo-Electron Microscopy and Molecular Dynamics Simulations to Investigate Membrane Binding of Influenza Virus Fusion Peptides

Supporting Information

Piotr Setny*[,1], Paulina Borkowska[2], Remigiusz Worch[2]
[1] Centre of New Technologies, University of Warsaw, Poland
[2] Nencki Institute of Experimental Biology Polish Academy of Sciences, Warsaw, Poland
**p.setny@cent.uw.edu.pl*

1 Liposome radii

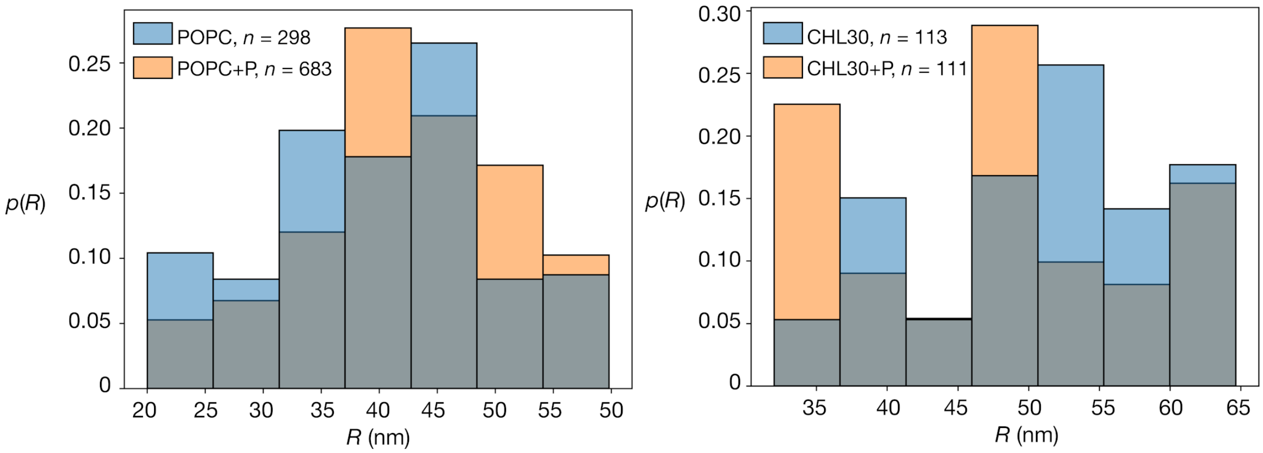

Figure 1: Distributions of liposome radii in experimental intensity profiles of POPC and CHL30 systems.

2 Simulated systems

Table 1: Number of lipid (both membrane leaflets) and peptide molecules in simulated systems, peptide to lipid ratios, simulation times, and numbers of simulation blocks used for independent synthetic intensity profiles generation.

conf.	POPC	CHL	peptides	$(P/L)\cdot 10^2$	t [μs]	blocks
POPC						
–	162	–	–	0	1.0	1
S	162	–	1	0.62	1.0	1
S	162	–	1	0.62	1.0	1
S	260	–	8	3.08	10.0	10
S	260	–	8	3.08	10.0	10
TM	162	–	1	0.62	1.0	1
TM	640	–	4	0.63	1.0	1
TM	540	–	8	1.48	3.3	3
TM	540	–	8	1.48	3.3	1
CHL30						
–	442	186	–	0	1.0	1
S	448	192	4	0.89	5.3	4
S	426	180	8	1.88	5.0	5
S	426	180	8	1.88	5.0	5
S	246	106	8	3.25	10.0	10
S	246	106	8	3.25	10.0	10
TM	162	74	1	0.62	1.0	1
TM	162	74	1	0.62	1.0	1
TM	456	192	8	1.75	3.9	3
TM	456	192	8	1.75	2.3	2

Note: simulations with multiple peptides in transmembrane configurations involved additional preliminary runs during which the centres of mass of the peptides were kept near membrane center plane ($z = 0$) using a flat bottom potential acting in z axis perpendicular to membrane surface. The potential was 0 for $z \in (-0.6, 0.6)$ nm, and smoothly switched to a harmonic potential with a force constant of 1000 kJ/mol/nm^2 for z outside this range. Accordingly, 1 s simulation blocks used for analysis extended over n last microseconds of production trajectory, leaving initial time for equilibration. These parts of trajectories were discarded from analysis and are indicated in respective plots in SI section 7.

3 Phase shift profile for planar bilayer-peptide system

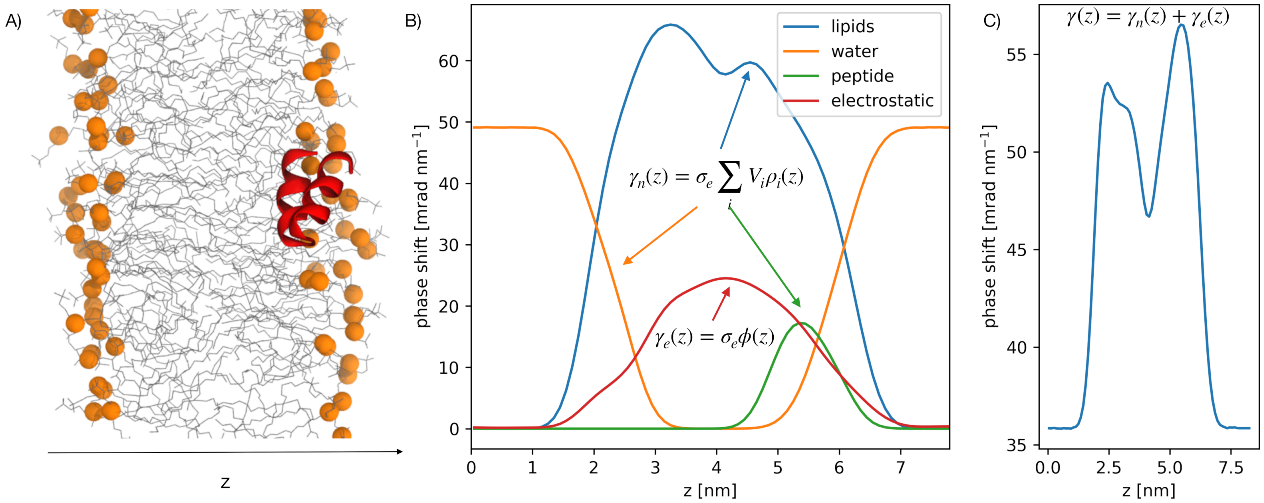

Figure 2: Phase shift profile for planar bilayer. A) Snapshot of simulated POPC membrane with peptide bound on the surface, B) components of phase shift profile: $V_i \rho_i(z)$ contributions from neutral atoms, $\phi(z)$ electrostatic potential, C) total phase shift profile.

4 Adjustment of calculated intensity amplitudes

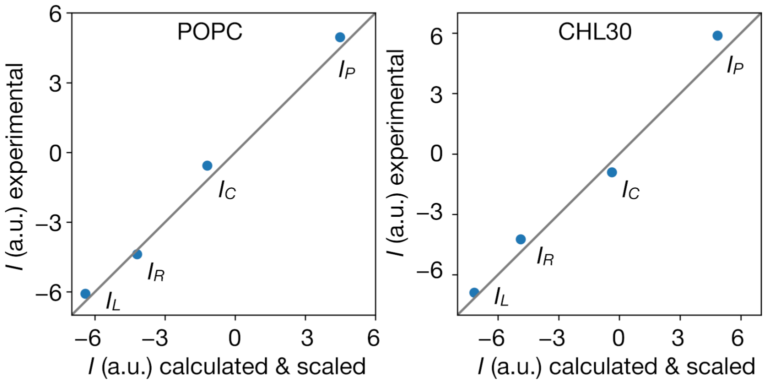

Figure 3: Linear dependence of experimental and calculated amplitudes of intensity profiles in POPC and CHL30 systems used to determine the scaling factors for calculated data.

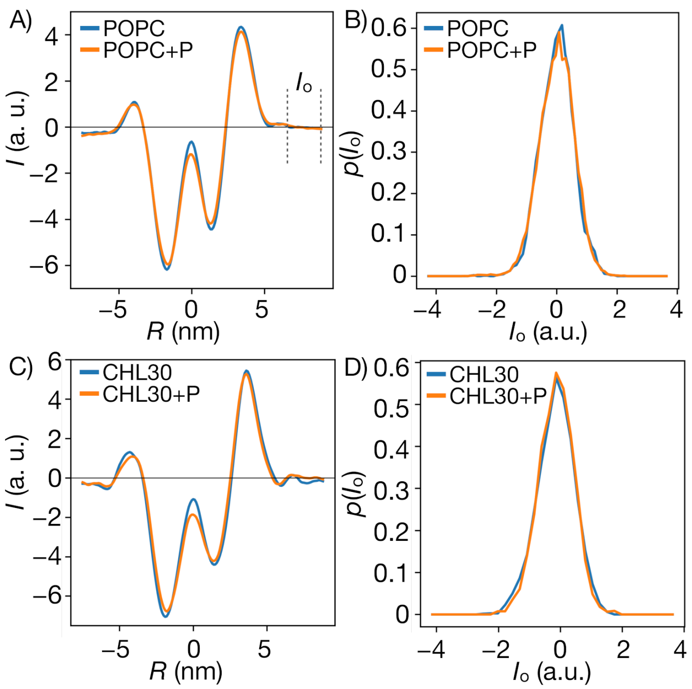

Figure 4: Experimental intensity profiles for pure and peptide-containing systems after amplitude adjustment. A,C) Profiles for POPC and CHL30 systems. B,D) Background (for $R \in (6, 9)$ nm from the central peak) intensity distributions in POPC and CHL30 systems. The distributions were compared for similarity using 2-sample Kolomogorov-Smirnov test, resulting in p-values of 0.15 and 0.24 for POPC and CHL30 systems, respectively.

5 Intensity profiles for cholesterol-containing liposomes

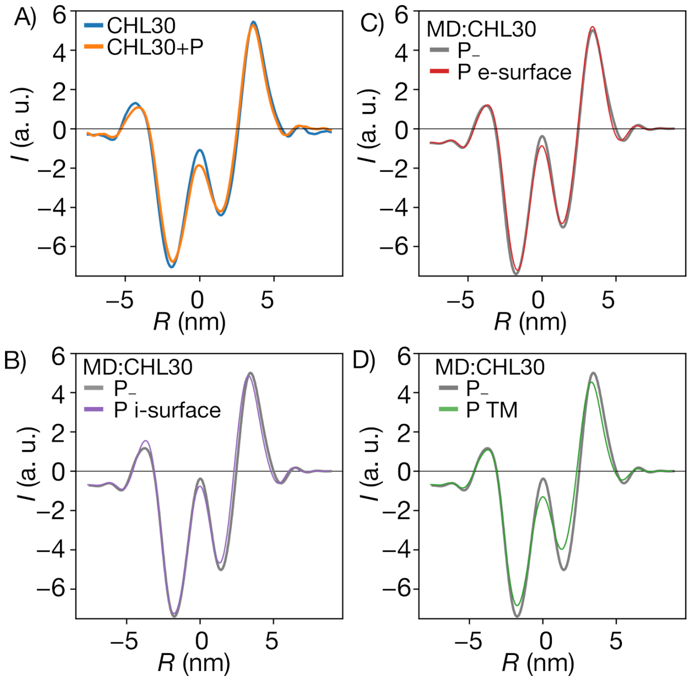

Figure 5: Experimental and simulated intensity profiles for CHL30 liposomes. A) experimental profiles for peptide-free and peptide-containing liposomes, B, C, D) simulated profiles for pure CHL30 membrane and three peptide binding modes.

6 Dependence of descriptor values on peptide concentration for cholesterol-containing systems

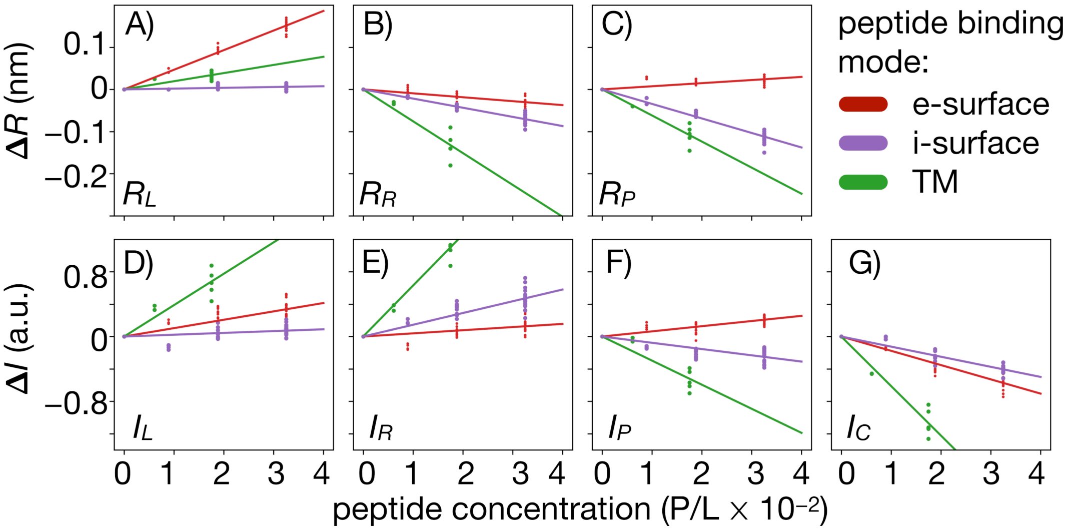

Figure 6: Changes in peak positions for increasing peptide concentration in CHL30 system; points: simulation data, lines: linear fits.

7 MD trajectories for multi-peptide simulations.

Below is a summary of MD trajectories. For multi-peptide simulations we indicate the occurrence of putative peptides aggregation. A figure for each such trajectory is organised in the following manner: **Left, upper plots:** positions of peptides centres of mass along the z axis, perpendicular to membrane plane. Dotted lines indicate mean positions of phosphorus atoms from each membrane leaflet. **Left, bottom panels:** the occurrence of peptide clusters containing $n \in \{1..4\}$ units. Single linkage clustering with 2.25 nm cutoff for distances in membrane plane (xy) between peptides centres of mass was used. The cutoff was determined based on the location of the first peak in radial distribution function of peptides centres of mass in xy plane, based on simulations depicted in SI figure 7. **Center plots:** cluster size statistics. **Right pictures:** final frames from trajectories.

POPC systems

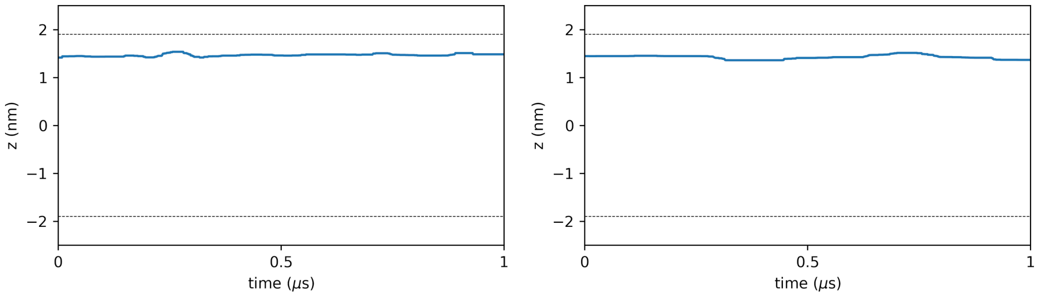

Figure 7: 1 peptide on the surface, 162 lipids, P/L= $0.62 \cdot 10^2$.

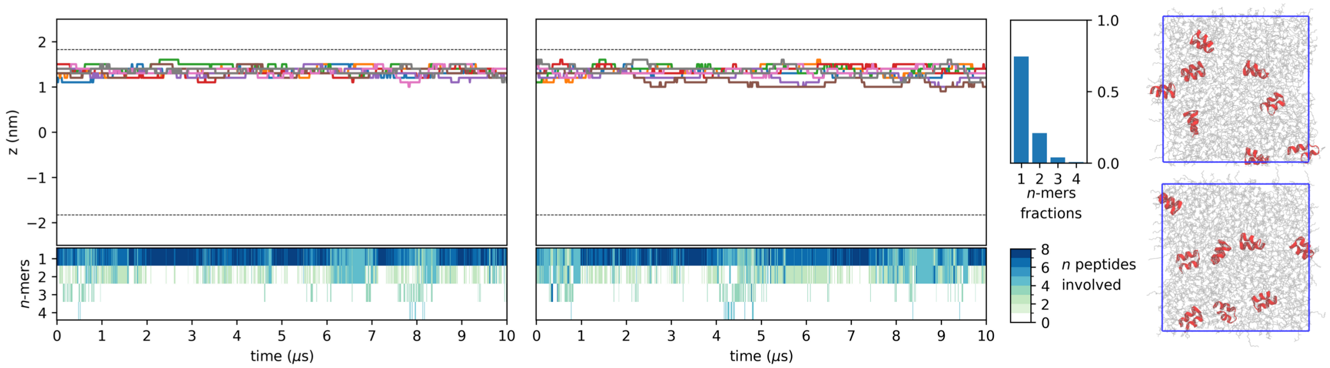

Figure 8: 8 peptides on the surface, 260 lipids, P/L= $3.08 \cdot 10^2$.

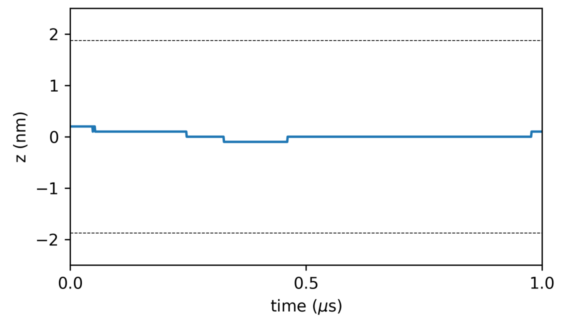

Figure 9: 1 transmembrane peptide, 162 lipids, P/L$= 0.62 \cdot 10^2$.

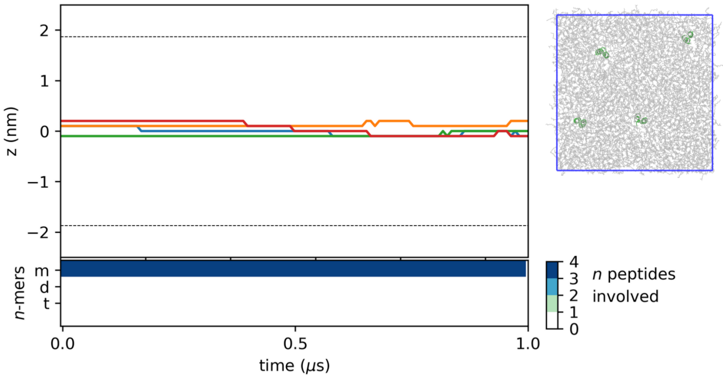

Figure 10: 4 transmembrane peptides, 640 lipids, P/L$= 0.63 \cdot 10^2$.

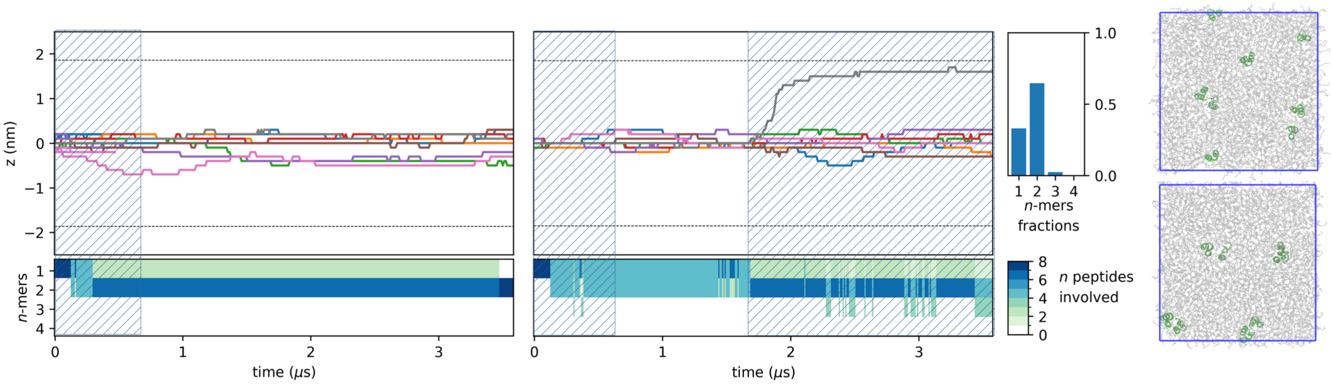

Figure 11: 8 transmembrane peptides, 540 lipids, P/L$= 1.48 \cdot 10^2$. Shaded trajectory segments were excluded from analysis.

CHL30 systems

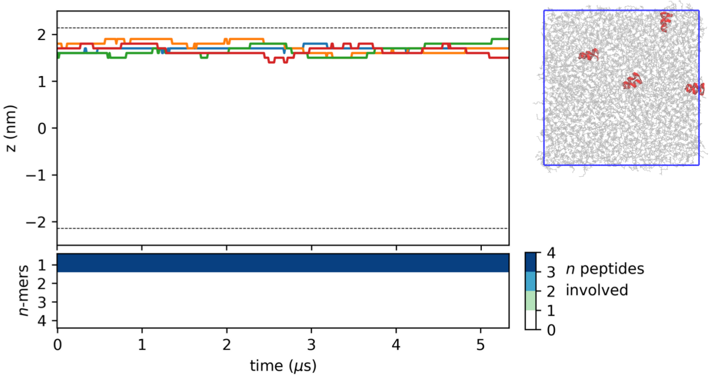

Figure 12: 4 peptides on the surface, 448 lipids, P/L= $0.89 \cdot 10^2$.

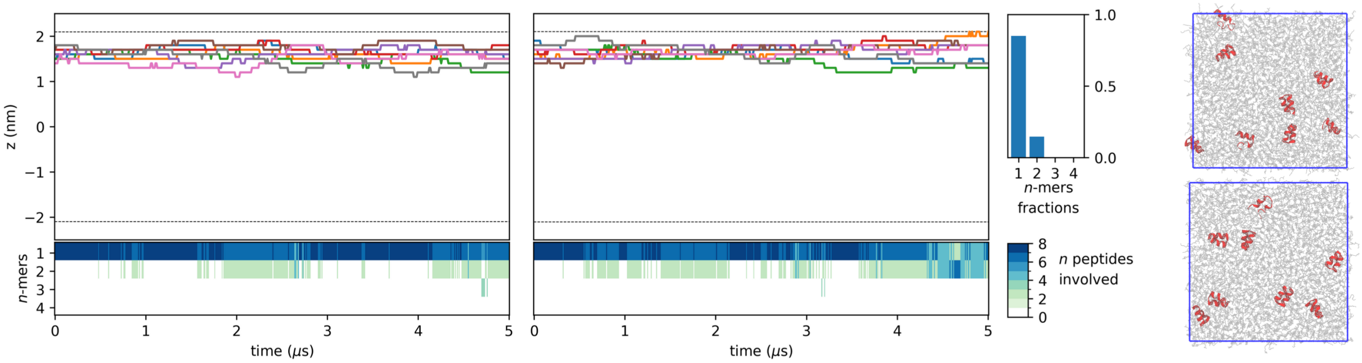

Figure 13: 8 peptides on the surface, 426 lipids, P/L= $1.88 \cdot 10^2$.

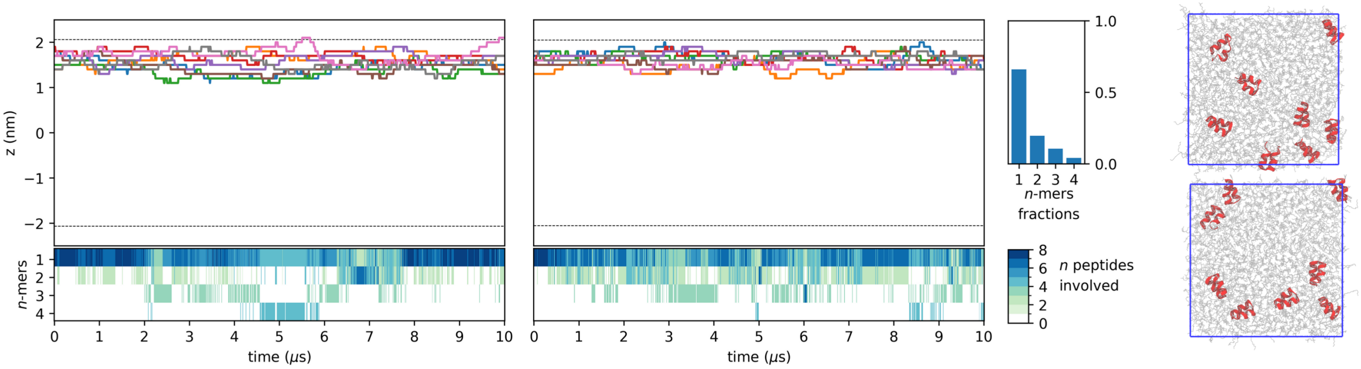

Figure 14: 8 peptides on the surface, 246 lipids, P/L$= 3.25 \cdot 10^2$.

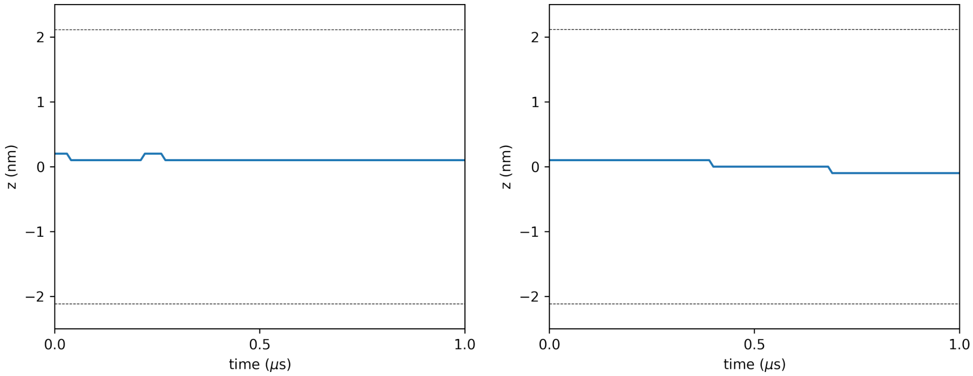

Figure 15: 1 transmembrane peptide, 162 lipids, P/L$= 0.62 \cdot 10^2$.

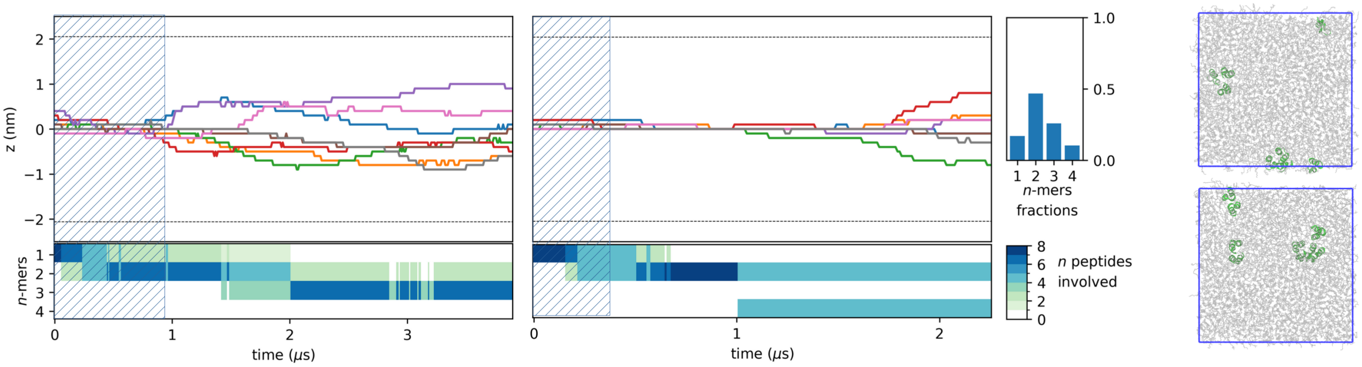

Figure 16: 8 transmembrane peptides, 456 lipids, P/L$= 1.75 \cdot 10^2$. Shaded trajectory segments were excluded from analysis.
